# A protocol for a systematic literature review: comparing the impact of seasonal and meteorological parameters on acute respiratory infections in Indigenous and non-Indigenous peoples

**DOI:** 10.1186/s13643-016-0399-x

**Published:** 2017-01-26

**Authors:** Katherine E. Bishop-Williams, Jan M. Sargeant, Lea Berrang-Ford, Victoria L. Edge, Ashlee Cunsolo, Sherilee L. Harper

**Affiliations:** 1 Department of Population Medicine, University of Guelph, 50 Stone Rd E, Guelph, ON N1G 2W1 Canada; 2 Centre for Public Health and Zoonoses, University of Guelph, Guelph, ON Canada; 3 Department of Geography, McGill University, Montreal, QC Canada; 4 Labrador Institute, Memorial University, Happy Valley-Goose Bay, NL Canada

**Keywords:** Systematic review protocol, Indigenous, Health, Season, Meteorological parameters, Respiratory infections, Climate, Weather

## Abstract

**Background:**

Acute respiratory infections (ARI) are a leading cause of morbidity and mortality globally, and are often linked to seasonal and/or meteorological conditions. Globally, Indigenous peoples may experience a different burden of ARI compared to non-Indigenous peoples. This protocol outlines our process for conducting a systematic review to investigate whether associations between ARI and seasonal or meteorological parameters differ between Indigenous and non-Indigenous groups residing in the same geographical region.

**Methodology:**

A search string will be used to search PubMed^®^, CAB Abstracts/CAB Direct^©^, and Science Citation Index^®^ aggregator databases. Articles will be screened using inclusion/exclusion criteria applied first at the title and abstract level, and then at the full article level by two independent reviewers. Articles maintained after full article screening will undergo risk of bias assessment and data will be extracted. Heterogeneity tests, meta-analysis, and forest and funnel plots will be used to synthesize the results of eligible studies.

**Discussion and registration:**

This protocol paper describes our systematic review methods to identify and analyze relevant ARI, season, and meteorological literature with robust reporting. The results are intended to improve our understanding of potential associations between seasonal and meteorological parameters and ARI and, if identified, whether this association varies by place, population, or other characteristics. The protocol is registered in the PROSPERO database (#38051).

**Electronic supplementary material:**

The online version of this article (doi:10.1186/s13643-016-0399-x) contains supplementary material, which is available to authorized users.

## Background

Acute respiratory infections (ARI) contribute to a substantial global burden of morbidity and mortality [1–3]. An estimated 14.9 million children were hospitalized for ARI in 2010, of which 265,000 died [3]. Defined as an acute infection with coughing as a symptom [4], ARI is often associated with meteorological parameters [1] and commonly varies by season [5]. For instance, ARI is commonly associated with temperature parameters, with increasing incidence during cold periods as a result of an individual’s exposure, susceptibility, and the infection type [6]. Furthermore, seasonal associations with ARI also have been identified; in some cases, these seasonal associations have been attributed to varying meteorological parameters and in other cases attributed to the pathogen’s own rhythmicity [6]. The associations between ARI and meteorological parameters and season may be modified by social gradients of health [7–9] and affected by type of livelihood [8, 10]. For instance, among Indigenous peoples, a strong connection to the land [11–13], resource-based livelihoods [14, 15], interacting social determinants of health (i.e., housing, education) [9, 14, 16], and the legacies of colonization [9] may modify the association between infections and meteorological and seasonal parameters [14]. Therefore, it is possible that there are differences in the association between weather variables and ARI among Indigenous and non-Indigenous communities. It is important to know if these associations differ between Indigenous and non-Indigenous communities to aid in better planning, resource allocation, and intervention strategies. This paper outlines a protocol for conducting a systematic review to investigate whether associations between ARI and seasonal or meteorological parameters differ between Indigenous and non-Indigenous groups residing in the same geographical region.

## Methods and design

This protocol, which outlines methods for the proposed systematic review, was designed in accordance with the Preferred Reporting Items for Systematic review and Meta-Analyses (PRISMA) Guidelines [17]. The protocol is registered in the PROSPERO database (#38051). The items of this protocol are presented in accordance with the PRISMA-P checklist (Additional file 1) [18].

## Review question

This systematic review protocol outlines the procedures for a systematic literature review that is intended to answer the question: Is the association between seasonal or meteorological parameters and ARI the same in Indigenous and non-Indigenous peoples who live in the same geographical region?

The components of population, exposure, comparator, and outcome (PECO) are as follows:

- Population: communities with Indigenous and non-Indigenous community members;
- Exposure: indigeneity;
- Comparator: non-Indigenous; and
- Outcome: association between seasonal or meteorological parameters and ARI.

## Study designs eligible

All primary epidemiological observational study designs (i.e., cross-sectional, cohort, case-control studies) are eligible for inclusion (Table 1). Ecological studies will be eligible, as the population in a singular location should be equally exposed to seasonal or meteorological parameters. Experimental studies (i.e., intervention studies) will not be eligible, as the exposure (i.e., indigeneity) cannot be assigned. Further, reviews, commentaries, editorials, mathematical models, or other non-primary research articles will not be eligible, as these studies are not comparable with observational results.

**Table 1:** Inclusion and exclusion criteria for a systematic literature review investigating the impact of seasonal and meteorological parameters on acute respiratory infection (ARI) in Indigenous and non-Indigenous peoples

Inclusion	Exclusion
The study is a primary epidemiological observational study design (cross-sectional, case-control, cohort, or ecological studies)	The study is an experiment (i.e., interventional), review, commentary, editorial, mathematical model, or other non-primary research
The research report exceeds 500 words	The research report is less than 500 words
The study’s population includes both Indigenous and non-Indigenous peoples	The study’s population includes only Indigenous or only non-Indigenous peoples
The study’s outcome is the association between ARI and seasonal or meteorological parameters	The study’s outcome is something other than the association between ARI and seasonal or meteorological parameters
The study provides sufficient epidemiological data to investigate the question of interest (i.e., two models representing the Indigenous and non-Indigenous strata separately with the same seasonal or meteorological parameter in both models, producing two measures of association for which the ratio of odds ratios (ROR) can be calculated, or (ii) a model for an ARI outcome that includes indigeneity and seasonal or meteorological parameter(s) as independent variables from which further calculations can be made)	The study provides insufficient information to investigate the question of interest
The study presents unique results which have not been previously published, or is the most recent and comprehensive analysis of the data.	The study which duplicates the results of a previous study

*ARI* acute respiratory infection

## Participants eligible

Eligible study populations are those in which a portion of the population living in the same region is explicitly defined as Indigenous and a portion of the population is explicitly defined as non-Indigenous. This research builds from the United Nations Declaration of the Rights of Indigenous Peoples [13] understanding of the term Indigenous peoples, which states that an Indigenous person self-identifies as Indigenous; has historical continuity with pre-colonial society; has a strong link to territory and natural resources; has a distinct social, economic, or political system; has a distinct language, culture, and/or belief system; forms a non-dominant societal group; and/or resolves to maintain and reproduce their ancestral environments and systems as distinctive peoples and societies.

## Exposures eligible

Indigeneity is the only eligible exposure. The comparator group is a non-Indigenous population living in the same region as the Indigenous population.

## Outcome measures eligible

**Fig. 1:**
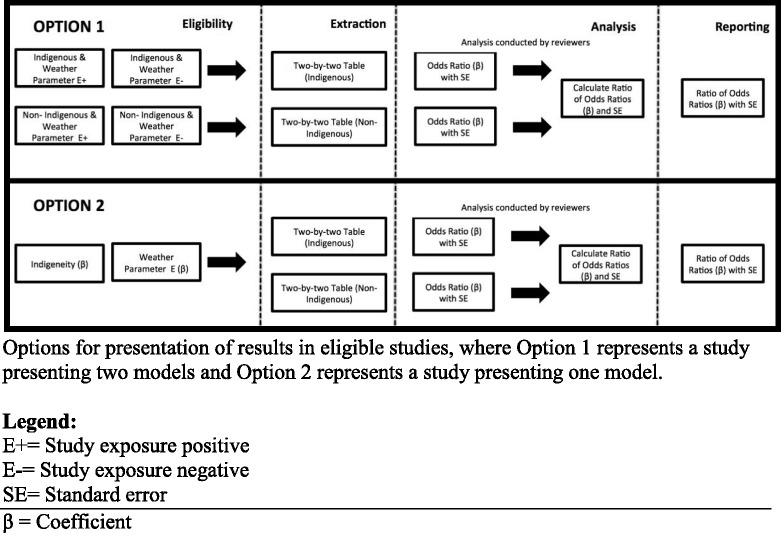
Options for presentation of results in eligible studies, where *Option 1* represents a study presenting two models and *Option 2* represents a study presenting one model. *E+* study exposure positive, *E-* study exposure negative, *SE* standard error, *β* coefficient

All eligible studies will present the association between ARI and weather variables. Eligible studies must investigate this association among both Indigenous and non-Indigenous peoples living in the same region. The results can be presented in one of two eligible ways: (i) two models representing the Indigenous and non-Indigenous strata separately with the same seasonal or meteorological parameter in both models, producing two measures of association for which the ratio of odds ratios (ROR) can be calculated (i.e., ARI-exposure model for Indigenous peoples and ARI-exposure model for non-Indigenous peoples); or (ii) a model for an ARI outcome that includes indigeneity and seasonal or meteorological parameter(s) as independent variables from which further calculations can be made (Fig. 1).

The case definition for ARI is an acute infection (i.e., less than 14 days duration, if duration is stated) with coughing as a primary symptom, with or without any accompanying symptoms. If duration is not provided in the case definition, the word “acute” will be sufficient for inclusion. The majority of ARI outcomes are anticipated to be respiratory syncytial virus, influenza, or pneumonia; however, studies reporting any ARI outcome are eligible. A diagnosis of ARI may include any symptomatic description that meets the case definition, a positive biological sample (e.g., swab), clinical diagnosis from a practitioner, or self-reported illness. Studies of non-infectious respiratory outcomes (e.g., asthma) are not eligible.

Seasonal variables refers to a pattern (i.e., season) in meteorological parameters related to a predictable trend such as temperature, hours of sunlight, or total precipitation that is repeated annually [19]. Meteorological parameters are defined as observable weather events, at a point in time, primarily consisting of temperature, precipitation, barometric pressure, humidity, sunlight, and the interactions and variability of these parameters [20]. In this review, any study that has seasonal or meteorological parameters measured at two or more points in time, and where the points are meteorologically or seasonally contrasting (i.e., measured in two different dates or in two different seasons), will be eligible.

## Search methods for the identification of studies

The search strategy comprises three main components: Indigenous communities (population and exposure terms); and association between seasonal or meteorological parameters and ARI (outcome terms; Table 2). Terms that will be used to identify Indigenous peoples globally are based on a series of umbrella terms for Indigenous used globally and throughout time, adapted from Bartlett et al. [21]. Individual group names were added to the umbrella terms from two sources. First, the International Work Group for Indigenous Affairs (IWGIA; www.iwgia.org) registry provides a continental directory of Indigenous peoples, further sorted as a country-by-country list of recognized Indigenous groups. These terms may be at the greater population level, rather than individual group level if the category can be expected to represent and include all of the unique peoples groups within it (i.e., the name “Maori” was included in the search terms, but individual Maori group names, such as Ngati Kuri, Ngati Maru, and To Arawa, were not included). Secondly, the United Nations Refugee Agency (UNHCR) provides a country-by-country database of minority and Indigenous peoples (www.refworld.org). Since this list provides both Indigenous and non-Indigenous peoples, only those groups explicitly listed as Indigenous were collected. The names of all identified groups defined as Indigenous were added to the search. When the two lists were complete, the lists were merged into one, alphabetized, and de-duplicated. The list of terms for Indigenous peoples is comprehensive to the best of the ability of this strategy. Searches of MeSH terms for “season,” “meteorology,” and “weather” were performed in PubMed^®^ and used to compile terms for the search strategy. Terms that will be used to identify ARI outcomes in the literature include any pathogen known to primarily cause ARI (i.e., enteric pathogens that rarely cause ARI are not included) based on the Medical Microbiology 4th Edition, Chapter 93, online version [22]. Any terms used for ARI by the Lung Disease Alphabetical Listing generated by the American Lung Association (www.lung.org) were added to the search strategy. A search of MeSH terms for “respiratory” and “lung infection” was performed in PubMed^®^ and any additional terms were added to the search strategy.

**Table 2:** Search string prepared for PubMed^®^

Population	Aasax OR Aboriginal OR “Aboriginal-Malay” OR Aborigine OR Achi OR Achuar OR Adibashi OR Adivasi OR Adivasis OR Afar OR Ainu OR Aka OR Akawai OR Akha OR Akie OR Akoula OR Akurio OR Akwoa OR “al-Kaabneh” OR “al-Asarmeh” OR “al-Ramadin” OR “al-Rshaida” OR “Alaska Native*” OR Aleut OR Alutor OR Amazigh OR Ambo OR “American Indian*” OR Ameridian OR Amuesha OR Anak OR “Andean Kichwe” OR Andoa OR Andorrans OR Angaite OR Anikhwe OR Anu OR Arara OR Arawak OR “Arawak-Taino” OR Arwak OR Ashaninka OR Atayal OR Austronesian OR “Ava Guarani” OR Awajun OR Awa OR Awakateco OR Aweer OR Ayeoreo OR Aymara OR Ayoreo OR Aztec OR Baaka OR Baantonu OR Babi OR Bahnar OR Babongo OR Bacwa OR Bagame OR Bagombe OR Bagyeli OR BaGyeli OR Bajuni OR Baka OR Bakgalagadi OR Bakola OR Bakongo OR Bakoya OR Balala OR Bambara OR Bambuti OR Bantu OR Barabaig OR Bariba OR Barimba OR Basarwa OR Bassari OR Batwa OR Bawarwa OR BaWka OR Bawn OR BaYeyi OR Bedzam OR Benet OR Berabis OR Berawan OR Berber OR Berbers OR Bidayuh OR Bigombe OR Biharis OR Bilma OR Bisayah OR Bobo OR Boeschs OR Bofi OR Boni OR Bonis OR Boranna OR Boro OR Bororo OR Boruka OR Botsarwa OR Bozo OR Brao OR Bribri OR “Bri Bri” OR Brunca OR Bugakhwe OR Bulu OR Bumipeuteras OR Bunak OR Bunun OR Bwiti OR Cabecar OR Cacaopera OR Campeche OR Carib OR Caribs OR “Ch’orti” OR Chachi OR Chaima OR Chakma OR Chalchiteco OR Chamorro OR Chamorru OR Chamorous OR “Chao-Khao” OR “Chao Ley” OR Charrua OR Chelkancy OR Chiapas OR Chibcha OR Chibchense OR Chipaya OR Chiriguano OR Chiquito OR Chiquitano OR Chorotega OR Chorti OR Cofan OR Chuaa OR Chuj OR Chukchi OR Chulymcy OR Chuvancy OR Ciboney OR “Ciboney-Taino-Arawak” OR “Cocama-Cocomilla” OR Colla OR Copts OR Cotier OR Cree OR Cumanagoto OR Dabou OR Dagiri OR Dahalo OR Danisi OR Daroobe OR Datoga OR Daza OR Degar OR Deti OR Diaguita OR Dinka OR Dioula OR Ditammari OR Dogon OR Dolgan OR Doma OR Dukha OR Dusun OR Ebrie OR “egga hodaabe” OR Elmolo OR “El Mono” OR Embera OR Emerillon OR Ency OR Endorois OR “Enlhet Norte” OR Enxet OR “Enxet Sur” OR Epera OR Eskimo OR “Ese Eja” OR Evenk OR Ewondo OR Fatukuku OR “First Nation*” OR “Forest dwell*” OR Fuegian OR Fulani OR Fulbe OR Galibia OR Galibi OR Garifuna OR Gaoshan OR Gio OR Guadalcanese OR Guana OR Guaicuru OR Guarani OR “Guarani Mbya” OR Guyami OR Guaymi OR Guerrero OR Gurani OR Guransi OR Gurung OR “G//ana” OR “G/wi” OR “Gwich’in” OR Hadzabe OR Hadza OR Haida OR Herero OR Hidalgo OR “Hill People” OR “Hill Person” OR Hmong OR Hoa OR Huambisa OR Huastec OR Hui OR Huetar OR Hutu OR Iban OR Igotot OR “Ik” OR Imazighn OR Imazighen OR Indigenous OR “Indigenous Palestinians” OR Ingarico OR Inuit OR Inupiat OR Inuvialut OR Iroquoian OR Itelmen OR “Itza’” OR Ixil OR Jacalteco OR “Jahalin Bedouin” OR Jarai OR Jivi OR Jumma OR “Ju’hoansi” OR “K’iche” OR “Ka Pei Aina” OR Kachin OR Kaiowa OR Kalanga OR Kalina OR “Kalina-go” OR Kalinago OR “Kalinago-Taino” OR “Kali’na” OR Kamchadal OR Kanak OR “Kanaka Maoli” OR Kanuri OR Kaqchikel OR Karamajong OR Karenni OR Kavalan OR Kayapo OR Kawashkar OR Kayan OR Kazakh OR Kedayan OR Kelait OR Kenyah OR Kereki OR Kety OR “Khali’nago” OR Khamu OR Khanty OR Khengs OR “Khmer Krom” OR Khoekhoe OR “Khoe-San” OR Khoikhoi OR Khoisan OR Khomani OR “Khudro Nrigoshthhi” OR Khumi OR Khwe OR Khyang OR Kichwas OR Kipsigis OR Kirdi OR Koba OR Koryak OR Krio OR Krohn OR Kua OR Kumandincy OR Kuna OR Kuy OR Kwisi OR Lahu OR Lao OR “Laotian Tribes” OR Lenca OR Lickanantay OR Limbu OR Lisu OR Livs OR Lobi OR Lokono OR Loma OR Lua OR Lumad OR “Lunda-Chokwe” OR Lushai OR Maasai OR Macourai OR Macuxi OR Macuzi OR Magar OR Makasae OR Makuxi OR Malagasy OR Malakote OR Malay OR “Malayo-Polynesian” OR Maleku OR Mangyan OR Mani OR Mano OR Mansi OR Maori OR Mam OR Manjo OR Marma OR “Marsh Dwellers” OR Mapuche OR Maskoy OR “masyarakat adat” OR Mataco OR “Mataco Matguayo” OR Matagulpa OR Maya OR “Maya Chorti” OR Mayagna OR Mbanderu OR Mbini OR Mbororo OR Mbukushu OR Mbundu OR Mbuti OR Mbri OR Mbya OR Mdendjele OR Melanesian OR “Melanesian-Papauan” OR Mestico OR Mestizo OR Merina OR Metis OR Miao OR Mien OR Mikaya OR Miskito OR Miskitu OR Misquito OR Mixte OR Mnong OR Mogeno OR “Mon-Khmer” OR Montagnards OR Mopan OR Moxeno OR Mozabite OR Mpukushu OR Mru OR Muong OR Murut OR “N/oakhwe” OR “N’guigmi” OR Nagas OR Nahoa OR Nahua OR Nahuatl OR Nama OR Nambiquara OR Nanaicy OR Nandeva OR “Nandevi Guarani” OR Naro OR “Naso Tjerdi” OR Native* OR “Native American*” OR “Native Hawai’ian*” OR Negidalcy OR Negeri OR Negrito OR Nemadi OR Nenets OR Nganasan OR Ngabe OR Ngobe OR “Ngobe-Bugle” OR Nivkhy OR Nuer OR “Nyaneka-Nkumbi” OR Oaxaca OR Ocanxiu OR Ogiek OR Ogoni OR Ojibway OR Okinawans OR “Orang Asli” OR Orochi OR Oroki OR Otomi OR Ovimibundu OR Oyampi OR “Pai Tavytera” OR Paiwan OR Palenqueros OR Palikur OR Pankho OR Patamona OR Pech OR Pemon OR Peul OR Peulh OR Penan OR Piaroa OR “Ping Pu” OR Pipil OR Pocomam OR Pokot OR Poqomam OR “Poqomchi’” OR Puebla OR Punan OR Puyuma OR “Q’anjob’al” OR “Q’eqchi” OR Qawasqar OR Qicaque OR Quechua OR Quenchua OR “Quintano Roo” OR Qom OR Rai OR Raisales OR Rakhine OR Rama OR Rapanui OR “Rapa Nui” OR Raute OR Rhade OR Roraima OR Rotumans OR Rukai OR Saami OR Sabaot OR Saharawis OR Saisyat OR Sakapulteco OR Sakizaya OR Sami OR (San AND Africa) OR Sanapan OR Sanapana OR Sandawe OR “Santa Rosa Carib” OR Sanya OR Saramancas OR (“Scheduled Tribes” AND India) OR Secoya OR Sediq OR Selkup OR Semang OR Sengwer OR Senoi OR Shan OR Sherpa OR Shipibo OR “Shipibo-Conibo” OR Shiwiar OR Shorcy OR Shua OR Shuar OR Siona OR Sipakapense OR Soioty OR “South Sea Islander*” OR Stieng OR “Sumu-Mayangna” OR Sutiaba OR Tachangya OR “Tai-Kadai” OR Taino OR “Taino-Kalingo” OR Tamang OR Tampuan OR Tapeba OR Tapebo OR Tareno OR Taurepang OR Tawahka OR Tazy OR Teda OR Teenek OR Teko OR Tektiteko OR Telengity OR Teleuty OR Temenbe OR Teribe OR Tesker OR Thakali OR Tharu OR Thao OR Tikuna OR Tikigaq OR Tirio OR Toba OR “Toba Maskoy” OR Tofolar OR Tolai OR Toloupan OR Tomarao OR Topnaars OR “Torres Straight Islander*” OR Totonac OR Toubou OR Truku OR Tsexakhwe OR Tripura OR Tsaatan OR Tsachila OR Tsou OR Tsumkwe OR Tshwa OR Tuareg OR Tuaregare OR Tubolar OR Tubu OR Tugen OR Tukano OR Tupi OR Tutong OR Tutsi OR “Tuvin-Todjin” OR Twa OR Tyua OR “Tz’utujil” OR Tzeltal OR Tzotzil OR Uchay OR Udege OR Ulchi OR “Ureueu-Wau-Wau” OR Uru OR Uspanteko OR Vadda OR Vadema OR Vai OR Veddhas OR Veps OR Vyadha OR “Waaniy-a-Laato” OR Waata OR Wadoma OR Wagashi OR Wapaichana OR Waorani OR Wapixana OR Warao OR Warrau OR “Warrau Wayana” OR Wayampi OR Wayana OR Wayeyi OR Wayuu OR Wichi OR Wodaabe OR Wounaan OR Xinka OR Yaaku OR Yami OR Yamana OR Yanomami OR Yukpa OR Yvytoso OR Zamuco OR Zapara OR Zapotec OR “!Xoo” OR “//’Xauesi” OR “/Xaisa” OR “‘Akateco” OR ((Indigenous OR Aboriginal OR Native) AND (Ache OR Algonquin OR Amis OR Bedouin OR Bugle OR Bushmen OR Dakota OR Dan OR Fang OR Herder OR Herdsmen OR Indian* OR Karen OR Maroon OR Mohawk OR Mon OR pastoralist* OR Papua OR Pear OR Potters OR Pygmy OR Pygymy OR Rade OR Roma OR Sab OR Squamish OR Tay OR Trio OR Yucatan))	The following terms were not found in PubMed^®^: Aboriginal-Malay, al-Asarmeh, al-Ramadin, al-Rshaida, Kichwe, chalchiteco, Chao-Khao, hodaabe, Enlhet, jivi, Ju'hoansi, kalina-go, Khali'nago, Khudro, Nrigoshthhi, Matguayo, oakhwe, Nandevi, Tjerdi, ocanxiu, Tavytera, Q'anjob'al, sakapulteco, sipakapense, Sumu-Mayangna, tikigaq, Maskoy, Tuvin-Todjin, tyua, ureueu-wau-wau, waaniy-a-laato, yvytoso, akateco.
Exposure	“atmospheric pressure” OR barometric OR cloud* OR cold OR “dew point” OR heat* OR humidity OR meteorolog* OR precipit* OR rain* OR season* OR snow* OR storm* OR sunshine OR temperature* OR “UV” OR “UV Index” OR “ultraviolet radiation” OR vapor OR warming OR weather OR wind OR winds OR windy	
Comparator	–	
Outcome (ARI and Pneumonia)	“acute chest syndrome” OR “acute respiratory distress syndrome” OR “ARDS” OR “bacterial pneumonia” OR “Black Lung Disease” OR bronchitis OR bronchiectasis OR bronchiolitis OR “bronchiolitis obliterans organizing pneumonia” OR “BOOP” OR “bronchopulmonary dysplasia” OR bronchopneumonia OR byssinosis OR “chest infection*” OR congest* OR coccidioidomycosis OR cocci OR cough* OR “cryptogenic organizing pneumonia” OR “flu” OR “Hantavirus Pulmonary Syndrome” OR histoplasmosis OR “Human Metapneumovirus” OR “Hypersensitivity Pneumonitis” OR Influenza* OR “lower respiratory tract infection” OR “LRTI” OR “LRTIs” OR “lung inflammation” OR “MERS” OR “Middle Eastern Respiratory Syndrome” OR “mycoplasma” OR “Non-tuberculosis Mycobacterium” OR pertussis OR pleuropneumonia OR pneumonconiosis OR pneumocystis OR pneumon* OR pneumophila OR “pneumocystis carnii” OR pulmonary OR Respiratory OR “Respiratory distress” OR “Respiratory distress syndrome” OR “respiratory tract infection*” OR “RTI” OR “RSV” OR “Respiratory Syncytial Virus” OR sarcoidosis OR “severe acute respiratory syndrome” OR streptococcus OR “tuberculosis” OR “TB” OR “upper respiratory tract infection*” OR “URTI” OR “URTIs” OR wheez* OR “viral pneumonia”	

All terms searched as “All Fields” terms. Collected 1259 citations on September 27, 2016

It is difficult to develop a search strategy that is robust enough to represent all nuances of the terms Indigenous, seasonal or meteorological parameters, and ARI, and thus, the use of multiple databases is intended to increase sensitivity of the search. This review will search the following databases: PubMed^®^ (via OvidSP^®^), CAB Abstracts/CAB Direct^©^, and Science Citation Index^®^ (via Web of Knowledge™). The search string will be appropriately adapted for each of the selected databases (Tables 2, 3, and 4). A university librarian was consulted in preparation of the search strategy for PubMed^®^.

**Table 3:** Search string prepared for Web of Knowledge™

Population	Aasax OR Aboriginal OR “Aboriginal-Malay” OR Aborigine OR Achi OR Achuar OR Adibashi OR Adivasi OR Adivasis OR Afar OR Ainu OR Aka OR Akawai OR Akha OR Akie OR Akoula OR Akurio OR Akwoa OR “al-Kaabneh” OR “al-Asarmeh” OR “al-Ramadin” OR “al-Rshaida” OR “Alaska Native*” OR Aleut OR Algonquin OR Alutor OR Amazigh OR Ambo OR “American Indian*” OR Ameridian OR Amuesha OR Anak OR “Andean Kichwe” OR Andoa OR Andorrans OR Angaite OR Anikhwe OR Anu OR Arara OR Arawak OR “Arawak-Taino” OR Arwak OR Ashaninka OR Atayal OR Austronesian OR “Ava Guarani” OR Awajun OR Awa OR Awakateco OR Aweer OR Ayeoreo OR Aymara OR Ayoreo OR Aztec OR Baaka OR Baantonu OR Babi OR Bahnar OR Babongo OR Bacwa OR Bagame OR Bagombe OR Bagyeli OR BaGyeli OR Bajuni OR Baka OR Bakgalagadi OR Bakola OR Bakongo OR Bakoya OR Balala OR Bambara OR Bambuti OR Bantu OR Barabaig OR Bariba OR Barimba OR Basarwa OR Bassari OR Batwa OR Bawarwa OR BaWka OR Bawn OR BaYeyi OR Bedouin OR Bedzam OR Benet OR Berabis OR Berawan OR Berber OR Berbers OR Bidayuh OR Bigombe OR Biharis OR Bilma OR Bisayah OR Bobo OR Boeschs OR Bofi OR Boni OR Bonis OR Boranna OR Boro OR Bororo OR Boruka OR Botsarwa OR Bozo OR Brao OR Bribri OR “Bri Bri” OR Brunca OR Bushmen OR Bugakhwe OR Bugle OR Bulu OR Bumipeuteras OR Bunak OR Bunun OR Bwiti OR Cabecar OR Cacaopera OR Campeche OR Carib OR Caribs OR “Ch’orti” OR Chachi OR Chaima OR Chakma OR Chalchiteco OR Chamorro OR Chamorru OR Chamorous OR “Chao-Khao” OR “Chao Ley” OR Charrua OR Chelkancy OR Chiapas OR Chibcha OR Chibchense OR Chipaya OR Chiriguano OR Chiquito OR Chiquitano OR Chorotega OR Chorti OR Cofan OR Chuaa OR Chuj OR Chukchi OR Chulymcy OR Chuvancy OR Ciboney OR “Ciboney-Taino-Arawak” OR “Cocama-Cocomilla” OR Colla OR Copts OR Cotier OR Cree OR Cumanagoto OR Dabou OR Dagiri OR Dahalo OR Dakota OR Danisi OR Daroobe OR Datoga OR Daza OR Degar OR Deti OR Diaguita OR Dinka OR Dioula OR Ditammari OR Dogon OR Dolgan OR Doma OR Dukha OR Dusun OR Ebrie OR “egga hodaabe” OR Elmolo OR “El Mono” OR Embera OR Emerillon OR Ency OR Endorois OR “Enlhet Norte” OR Enxet OR “Enxet Sur” OR Epera OR Eskimo OR “Ese Eja” OR Evenk OR Ewondo OR Fatukuku OR “First Nation*” OR “Forest dwell*” OR Fuegian Fulani OR Fulbe OR Galibia OR Galibi OR Garifuna OR Gaoshan OR Gio OR Guadalcanese OR Guana OR Guaicuru OR Guarani OR “Guarani Mbya” OR Guyami OR Guaymi OR Guerrero OR Gurani OR Guransi OR Gurung OR “G//ana” OR “G/wi” OR “Gwich’in” OR Hadzabe OR Hadza OR Haida OR Herder OR Herdsmen OR Herero OR Hidalgo OR “Hill People” OR “Hill Person” OR Hmong OR Hoa OR Huambisa OR Huastec OR Hui OR Huetar OR Hutu OR Iban OR Igotot OR “Ik” OR Imazighn OR Imazighen OR Indigenous OR “Indigenous Palestinians” OR Ingarico OR Inuit OR Inupiat OR Inuvialut OR Iroquoian OR Itelmen OR “Itza’” OR Ixil OR Jacalteco OR “Jahalin Bedouin” OR Jarai OR Jivi OR Jumma OR “Ju’hoansi” OR “K’iche” OR “Ka Pei Aina” OR Kachin OR Kaiowa OR Kalanga OR Kalina OR “Kalina-go” OR Kalinago OR “Kalinago-Taino” OR “Kali’na” OR Kamchadal OR Kanak OR “Kanaka Maoli” OR Kanuri OR Kaqchikel OR Karamajong OR Karenni OR Kavalan OR Kayapo OR Kawashkar OR Kayan OR Kazakh OR Kedayan OR Kelait OR Kenyah OR Kereki OR Kety OR “Khali’nago” OR Khamu OR Khanty OR Khengs OR “Khmer Krom” OR Khoekhoe OR “Khoe-San” OR Khoikhoi OR Khoisan OR Khomani OR “Khudro Nrigoshthhi” OR Khumi OR Khwe OR Khyang OR Kichwas OR Kipsigis OR Kirdi OR Koba OR Koryak OR Krio OR Krohn OR Kua OR Kumandincy OR Kuna OR Kuy OR Kwisi OR Lahu OR Lao OR “Laotian Tribes” OR Lenca OR Lickanantay OR Limbu OR Lisu OR Livs OR Lobi OR Lokono OR Loma OR Lua OR Lumad OR “Lunda-Chokwe” OR Lushai OR Maasai OR Macourai OR Macuxi OR Macuzi OR Magar OR Makasae OR Makuxi OR Malagasy OR Malakote OR Malay OR “Malayo-Polynesian” OR Maleku OR Mangyan OR Mani OR Mano OR Mansi OR Maori OR Mam OR Manjo OR Marma OR “Marsh Dwellers” OR Mapuche OR Maskoy OR “masyarakat adat” OR Mataco OR “Mataco Matguayo” OR Matagulpa OR Maya OR “Maya Chorti” OR Mayagna OR Mbanderu OR Mbini OR Mbororo OR Mbukushu OR Mbundu OR Mbuti OR Mbri OR Mbya OR Mdendjele OR Melanesian OR “Melanesian-Papauan” OR Mestico OR Mestizo OR Merina OR Metis OR Miao OR Mien OR Mikaya OR Miskito OR Miskitu OR Misquito OR Mixte OR Mnong OR Mogeno OR Mohawk OR Mon OR “Mon-Khmer” OR Montagnards OR Mopan OR Moxeno OR Mozabite OR Mpukushu OR Mru OR Muong OR Murut OR “N/oakhwe” OR “N’guigmi” OR Nagas OR Nahoa OR Nahua OR Nahuatl OR Nama OR Nambiquara OR Nanaicy OR Nandeva OR “Nandevi Guarani” OR Naro OR “Naso Tjerdi” OR Native* OR “Native American*” OR “Native Hawai’ian*” OR Negidalcy OR Negeri OR Negrito OR Nemadi OR Nenets OR Nganasan OR Ngabe OR Ngobe OR “Ngobe-Bugle” OR Nivkhy OR Nuer OR “Nyaneka-Nkumbi” OR Oaxaca OR Ocanxiu OR Ogiek OR Ogoni OR Ojibway OR Okinawans OR “Orang Asli” OR Orochi OR Oroki OR Otomi OR Ovimibundu OR Oyampi OR “Pai Tavytera” OR Paiwan OR Palenqueros OR Palikur OR Pankho OR Papua OR Patamona OR Pech OR Pemon OR Peul OR Peulh OR Penan OR Piaroa OR “Ping Pu” OR Pipil OR Pocomam OR Pokot OR Poqomam OR “Poqomchi’” OR Puebla OR Punan OR Puyuma OR “Q’anjob’al” OR “Q’eqchi” OR Qawasqar OR Qicaque OR Quechua OR Quenchua OR “Quintano Roo” OR Qom OR Rai OR Raisales OR Rakhine OR Rama OR Rapanui OR “Rapa Nui” OR Raute OR Rhade OR Roraima OR Rotumans OR Rukai OR Saami OR Sab OR Sabaot OR Saharawis OR Saisyat OR Sakapulteco OR Sakizaya OR Sami OR (San AND Africa) OR Sanapan OR Sanapana OR Sandawe OR “Santa Rosa Carib” OR Sanya OR Saramancas OR Scheduled Tribes OR Secoya OR Sediq OR Selkup OR Semang OR Sengwer OR Senoi OR Shan OR Sherpa OR Shipibo OR “Shipibo-Conibo” OR Shiwiar OR Shorcy OR Shua OR Shuar OR Siona OR Sipakapense OR Soioty OR “South Sea Islander*” OR Stieng OR “Sumu-Mayangna” OR Sutiaba OR Tachangya OR “Tai-Kadai” OR Taino OR “Taino-Kalingo” OR Tamang OR Tampuan OR Tapeba OR Tapebo OR Tareno OR Taurepang OR Tawahka OR Tazy OR Teda OR Teenek OR Teko OR Tektiteko OR Telengity OR Teleuty OR Temenbe OR Teribe OR Tesker OR Thakali OR Tharu OR Thao OR Tikuna OR Tikigaq OR Tirio OR Toba OR “Toba Maskoy” OR Tofolar OR Tolai OR Toloupan OR Tomarao OR Topnaars OR “Torres Straight Islander*” OR Totonac OR Toubou OR Truku OR Tsexakhwe OR Tripura OR Tsaatan OR Tsachila OR Tsou OR Tsumkwe OR Tshwa OR Tuareg OR Tuaregare OR Tubolar OR Tubu OR Tugen OR Tukano OR Tupi OR Tutong OR Tutsi OR “Tuvin-Todjin” OR Twa OR Tyua OR “Tz’utujil” OR Tzeltal OR Tzotzil OR Uchay OR Udege OR Ulchi OR “Ureueu-Wau-Wau” OR Uru OR Uspanteko OR Vadda OR Vadema OR Vai OR Veddhas OR Veps OR Vyadha OR “Waaniy-a-Laato” OR Waata OR Wadoma OR Wagashi OR Wapaichana OR Waorani OR Wapixana OR Warao OR Warrau OR “Warrau Wayana” OR Wayampi OR Wayana OR Wayeyi OR Wayuu OR Wichi OR Wodaabe OR Wounaan OR Xinka OR Yaaku OR Yami OR Yamana OR Yanomami OR Yucatan OR Yukpa OR Yvytoso OR Zamuco OR Zapara OR Zapotec OR !Xoo OR//’Xauesi OR/Xaisa OR ‘Akateco OR ((Indigenous OR Aboriginal OR Native) AND (Ache OR Amis OR Dan OR Fang OR Indian* OR Karen OR Maroon OR pastoralist* OR Pear OR Potters OR Pygmy OR Pygymy OR Rade OR Roma OR Squamish OR Tay OR Trio))
Exposure	“atmospheric pressure” OR barometric OR cloud* OR cold OR “dew point” OR heat* OR humidity OR meteorolog* OR precipit* OR rain* OR season* OR snow* OR storm* OR sunshine OR temperature* OR “UV” OR “UV Index” OR “ultraviolet radiation” OR vapor OR warming OR weather OR wind OR winds OR windy
Comparator	-
Outcome (ARI and Pneumonia)	“acute chest syndrome” OR “acute respiratory distress syndrome” OR “ARDS” OR “bacterial pneumonia” OR “Black Lung Disease” OR bronchitis OR bronchiectasis OR bronchiolitis OR “bronchiolitis obliterans organizing pneumonia” OR “BOOP” OR “bronchopulmonary dysplasia” OR bronchopneumonia OR byssinosis OR “chest infection*” OR congest* OR coccidioidomycosis OR cocci OR cough* OR “cryptogenic organizing pneumonia” OR “flu” OR “Hantavirus Pulmonary Syndrome” OR histoplasmosis OR “Human Metapneumovirus” OR “Hypersensitivity Pneumonitis” OR Influenza* OR “lower respiratory tract infection” OR “LRTI” OR “LRTIs” OR “lung inflammation” OR “MERS” OR “Middle Eastern Respiratory Syndrome” OR “mycoplasma” OR “Non-tuberculosis Mycobacterium” OR pertussis OR pleuropneumonia OR pneumonconiosis OR pneumocystis OR pneumon* OR pneumophila OR “pneumocystis carnii” OR pulmonary OR Respiratory OR “Respiratory distress” OR “Respiratory distress syndrome” OR “respiratory tract infection*” OR “RTI” OR “RSV” OR “Respiratory Syncytial Virus” OR sarcoidosis OR “severe acute respiratory syndrome” OR streptococcus OR “tuberculosis” OR “TB” OR “upper respiratory tract infection*” OR “URTI” OR “URTIs” OR wheez* OR “viral pneumonia”

All terms searched as “Topic” terms. Collected 1475 citations on September 26, 2016

**Table 4:** Search string prepared for CAB Abstracts/CAB Direct^©^

Population	Indigenous OR Aborigine OR Aboriginal OR Native OR Indian OR Tribe OR “Tribal Group”	Globally and temporally inclusive terms for Indigenous only (no group names).
Exposure	“atmospheric pressure” OR barometric OR cloud* OR cold OR “dew point” OR heat* OR humidity OR meteorolog* OR precipit* OR rain* OR season* OR snow* OR storm* OR sunshine OR temperature* OR “UV” OR “ultraviolet radiation” OR vapor OR warming OR weather OR wind OR winds OR windy	No changes made from full search term list
Comparator	-	
Outcome (ARI and Pneumonia)	bronchitis OR bronchiectasis OR bronchiolitis OR “chest infection*” OR congest* OR cough* OR “flu” OR Influenza* OR “lung inflammation” OR “Middle Eastern Respiratory Syndrome” OR “mycoplasma” OR pertussis OR pneumon* OR pulmonary OR Respiratory OR “Respiratory Syncytial Virus” OR streptococcus OR wheez*	Removed all acronyms, maintained symptoms and key diseases (more generic names only)

All terms searched as “Title” or “Abstract” or “Subject.” Collected 207 citations on September 27, 2016

The search will not be limited by language, date, or study design. Search terms will be in English, although the names of Indigenous groups are commonly stated in their own languages (i.e., non-English names in the roman alphabet syntax will be used). An English search string should identify all English articles and any non-English articles with an English title and abstract. If a non-English citation is collected by the search, Google Translate^©^ will be used to translate the title and abstract for initial screening [23], and if maintained for full article screening, Google Translate^©^ will be used for full text screening. This will allow calculation of the total number of eligible articles to generate appropriate denominators. If after full article screening a non-English article is eligible, the article will be formally translated by a paid service, if funding is available. If it is not possible to translate non-English articles, they will be excluded from data extraction and risk of bias (i.e., after full-text screening).

To minimize the risk of exclusion of relevant citations, the citation list of each included study will be searched (i.e., a snowball search). Additionally, Google Scholar^©^ will be used to complete a citation search on eligible studies, to identify studies that have referenced these studies. Studies identified by either the hand-searches or citation searches will be screened for relevance.

Published and unpublished literature will be eligible. Published literature can be collected by all of the proposed databases. Unpublished literature can be collected by CAB Abstract/CAB Direct^©^ and Science Citation Index^®^. Unpublished primary research that exceeds 500 words in length will be eligible if it meets one of the three following criteria: (i) governmental report (i.e., produced by a regional or national government ministry); (ii) non-governmental report (i.e., produced by non-governmental organizations); or (iii) graduate or honor undergraduate thesis or dissertation. Reports that are fewer than 500 words will be excluded.

If the initial search identifies any relevant government or non-governmental reports, or theses, a search will be conducted in PubMed^®^ to identify any relevant journal publications by the first author of the citation. This PubMed^®^ search will include the first author’s last name and first initial, institutional affiliation, and one to three keywords from the abstract (i.e., author identified key words if available, or reviewer key words if not available).

To keep the review current, if more than 12 months pass from the date the search was conducted to completion of data extraction and analysis, an update search will be conducted. If conducted, the second search will use the same search strategy as the first and will not be restricted by date. The search will be conducted in all of the original databases. Thus, a recall strategy is employed [24], which should identify all of the initial studies and all studies published since the previous search. A hand-matching method will be used to identify whether all of the original citations are included.

## Selection of eligible studies

All search result citations will be loaded into and managed in EndNote™ bibliographic software and de-duplicated automatically. Then, citations will be uploaded from EndNote™ into DistillerSR^®^, which will be used for form generation, screening, and management of relevant screening level statistics.

Screening will be completed in two stages. Screening processes will be piloted and tested by the reviewers on a subset of studies (5% of studies if *n* > 50, 10% of studies if *n* ≤ 50). First, title and abstract screening will be conducted on all citations identified. Two reviewers with graduate-level training in epidemiology and systematic literature review processes will screen articles independently, using five evaluation questions (Table 5). All questions will be answered as “yes,” “no,” or “unsure.” In this screening phase, questions will be hidden. A hidden question will not be answered if the article is excluded based on previous screening questions. Articles will be excluded if both reviewers answer “no” to any of the five questions. If both reviewers answer “yes” and/or “unsure” to all questions it will be maintained for full article screening. Any disagreements will be resolved by consensus. When consensus cannot be reached, a third reviewer will arbitrate.

**Table 5:** Title and abstract screening questions to be used to identify literature for inclusion in the full article screening process

Screening domain and question	Characteristics for assessment		
	Yes—include	No—exclude	Unclear—include
Research design: Does the title/abstract describe a primary observational research study?	Yes: The study employs an observational or ecological study design.	No: The study is a review, commentary, editorial, mathematical model, or other non-primary research, or is experimental.	Unclear: It is unclear if the study design is primary research from the title and abstract.
Publication type: Does the title and abstract come from a published study, government report, non-governmental report, or post-secondary institutional thesis (exceeding 500 words in length)?	Yes: The study is a published study, or government report, non-governmental report, or post-secondary institutional thesis (exceeding 500 words in length).	No: The study is not a published study, government report, non-governmental report, or post-secondary institutional thesis or is fewer than 500 words in length.	Unclear: It is unclear if the study is a published study, or government report, non-governmental report, or post-secondary institutional thesis (exceeding 500 words in length) from the title and abstract.
Population: Does the population of interest include both an Indigenous population and non-Indigenous population living in the same geographical region?	Yes: The study population describes both Indigenous and non-Indigenous peoples living in the same geographical region.	No: The population of interest is entirely Indigenous or non-Indigenous or the populations are not within the same region.	Unclear: It is unclear if the population of interest is both Indigenous and non-Indigenous from the title and abstract.
Outcome: Does the title and/or abstract describe research on the association between ARI and any exposure?	Yes: One or all of the outcomes of interest in the study are ARI, defined by the case definition for this review (i.e. acute infection (less than 14 days duration, if duration is stated) with coughing as symptom, with or without any accompanying symptoms).	No: There is no ARI-related health outcome measured in the study.	Unclear: It is unclear if the health outcome of interest is ARI from the title and abstract.
Exposure: Is the study’s exposure of interest a seasonal or meteorological factor (defined as observable weather events, primarily consisting of temperature, precipitation, barometric pressure, humidity, sunlight, and the interactions and temporal variability of these parameters)?	Yes: The exposure of interest is a seasonal or meteorological factor.	No: The exposure of interest is not a seasonal or meteorological factor.	Unclear: It is unclear if the exposure of interest is a seasonal or meteorological factor.

Second, full article screening will be conducted on all citations remaining after title and abstract screening. Two reviewers will screen articles independently, using a second form in DistillerSR^®^. Reviewers will use seven evaluation questions (Table 6). In full article screening, reviewers will identify any studies using a duplicate dataset and will maintain the research that is most comprehensive. Duplicate results will be removed. Questions will not be hidden in full article screening, allowing for analysis of the reason for exclusion. Studies will be excluded if both reviewers answer “no” to any question. Disagreements between reviewers will be resolved by consensus and, when consensus cannot be reached, a third reviewer will arbitrate.

**Table 6:** Full article screening questions to be used to identify literature for inclusion

Screening domain and question	Characteristics for assessment	
	Yes—include	No—exclude
Comparator: Does the research compare the association between seasonal or meteorological parameters and ARI between Indigenous and non-Indigenous groups (either via two models or a model that accounts for Indigeneity).	Yes: The research compares associations between seasonal or meteorological parameters and ARI in the context of Indigeneity.	No: The research does not compare associations between seasonal or meteorological parameters and ARI in the context of Indigeneity.
Duplicates: Does the research use a new dataset for analysis (i.e., the dataset was not previously analyzed in another included study).	Yes: The research uses a new dataset for analysis.	No: The research uses a dataset that has been previously analyzed by another included study. (Note: When identifying the study to be included, the most comprehensive study will be used.)

Full article screening questions will include all screening domains and questions from title and abstract screening as well as two additional questions

## Data collection from eligible studies

Data extraction will include study identifiers and study design; participant, exposure, and outcome information; and information about analytical methods. Missing information will be noted.

Extracted study identifiers will be the authors’ names; study title; publication type; publication date; journal, volume, issue, and page numbers of publication; place of publication (i.e., first author’s institutional address); and digital object identifier. Study design, time frame of study, climate zone of interest, location of study (i.e., country), and region of study (localized when reported) will also be extracted.

Data extracted about participants will include the definition of the target and source populations, size of the target population, and size of the source population. Relevant demographic information (e.g., age, sex) at the study population level will be extracted when reported.

Exposure related data extracted will include the name of the Indigenous population, the size of the Indigenous source population, and the size of the Indigenous study population. For the comparator, the size of the non-Indigenous source population, and the size of the non-Indigenous study population will be extracted. For all studies, the definition provided for Indigenous peoples will be extracted. Further, if causal mechanisms for differences in seasonal or meteorological effects on ARI are provided, these will be extracted.

Information related to the seasonal or meteorological parameter(s) of interest, as well as the ARI outcome(s) of interest will be extracted. In addition, the association(s) between ARI and season or meteorological parameters will be extracted. For season and meteorological parameters, extracted information will include the name of each parameter (e.g., rainfall) and its related measure (e.g., millimeters); type of temporal cycle (e.g., daily, seasonal, annual); and number of cycles completed (e.g., years). Additionally, the source of data used to evaluate the season or meteorological parameter will be collected (e.g., meteorological stations). The extracted information for each ARI outcome will be the specific ARI outcome (i.e., case definition), measurement of the ARI outcome (e.g., self-reported), group-level metric for each population group (e.g., prevalence) and the effect size (i.e., beta) comparing the Indigenous and non-Indigenous peoples or strata (e.g., odds ratio). Where two models are presented (e.g., ARI-exposure models for Indigenous and non-Indigenous peoples), odds ratios will be extracted for each strata (Fig. 1). Where one model is presented (e.g., a model with Indigeneity and weather parameter(s) as fixed effects), the regression coefficients for the season or meteorological parameter and indigeneity will be extracted. In the case that a study presents results for both options (i.e., two strata models and a single model with fixed effects), data will be extracted for both options. For each association, the measure(s) of precision (e.g., standard error of the mean, standard deviation, and/or confidence intervals) will be extracted when provided. If only the *p* value and sample size are reported, these data will be extracted and a measure of precision will be calculated from the available data for each association.

Finally, information will be extracted on the type of modeling or statistical approach (e.g., linear regression) used, and if and which confounders were considered. Confounders considered will be extracted and a list will be generated for various ARI outcomes. Additionally, the unit of analysis (e.g., individual, household, or community) and spatial resolution of the climate data used for modeling will be extracted.

## Process for data extraction

A data extraction form will be created in DistillerSR^®^. The extraction form will be piloted and tested by the data extractors on a subset of studies (5% of studies if *n* > 50, 10% of studies if *n* ≤ 50). Following pilot testing, the form will be adapted as recommended by the extractors to improve usability and completeness. The first author and one additional extractor who each have training in epidemiology and systematic literature review processes will complete data extraction. Data extraction will be completed independently and the extractors will compare the data for consensus. If the extractors cannot answer a question, consensus will confirm that the data are unavailable to answer the question.

In the event that the data presented in a study are unclear, missing, or presented in a non-extractable or unusable form, authors of studies published in the last 5 years (since January 1, 2011) will be contacted for clarification. Authors will be contacted via email, and a follow-up email will be sent 2 weeks later. Authors will be provided 4 weeks from the initial contact to respond. If data from older studies are unclear, missing, or presented in a non-extractable or unusable form, authors will not be contacted. Missing data will be noted in the report.

## Risk of bias assessment for eligible studies

The risk of bias (ROB) assessment was adjusted from existing tools (i.e., risk of bias in non-randomized studies of interventions) [25]. In particular, adjustments were needed to account for ecological studies and repeated measures. One question was added to investigate ROB due to ecological studies and one question was added to investigate ROB due to repeated measures. Questions related to experimental interventions were removed. Two reviewers will conduct the ROB assessment independently, in conjunction with data extraction, at the study level (i.e., one ROB analysis will be conducted per study). Both reviewers conducting the ROB assessment will have advanced graduate training in epidemiology and bias assessment. In total, nine domains of bias will be tested according to predetermined criteria for high, low, or unclear ROB. The ROB will be conducted using a form in DistillerSR^®^ with a textbox to record the rationale for selecting the level of ROB for each domain.

## Confounders relevant to all or most studies

Since this review will focus on the association between weather parameters and ARI outcomes, confounders in this study are those that affect the association between these weather parameters and ARI in Indigenous versus non-Indigenous peoples. Important confounders that could affect all or most studies are: (i) gender, (ii) age, and (iii) local wealth (e.g., regional or national).

## Strategy for data synthesis

The review will analyze information for both associations (systematic review and meta-analyses) and context (e.g., via descriptive statistics, narrative, and descriptive spatial analyses). This approach is necessary to avoid comparing unlike populations, exposures, or outcomes. Analyses will be completed using STATA^©^ version 13.1 and REVMAN^©^ version 5.

Prior to beginning meta-analyses, descriptive statistics will be conducted on extracted data. Frequencies, proportions, and missing data will be considered for each extracted variable. The descriptive data will serve to describe the literature available on this topic and to represent the dataset under study.

Meta-analyses will be conducted for each ARI association (i.e., each ARI outcome and weather parameter identified) that has at least two studies providing data (i.e., two studies presenting the association between the same ARI outcome and same weather parameter). The outcome used for meta-analyses will be the ROR representing the relative effects of weather parameters on ARI between Indigenous and non-Indigenous peoples (Fig. 1). For studies presenting two models (i.e., ARI associations for each strata), odds ratios for each eligible study for each group will be extracted (i.e., Indigenous, non-Indigenous) to calculate a ROR. In studies presenting a single model, regression coefficients will be used directly to calculate odds ratios and solve for the ROR (Fig. 1). The standard error of the ROR will be calculated according to Golder et al. [26] for both study types. Meta-analyses will be conducted using random effects models.

Between-study heterogeneity will be explored using the *I* ^2^ statistic (*I* ^2^ < 0.25 considered homogeneous). If heterogeneity exists, sources of heterogeneity will be explored by sub-group analyses. For eligible studies, we propose to categorize the studies by (i) population, (ii) outcome, (iii) exposure, and (iv) location. Studies will be categorized by groups of Indigenous peoples (e.g., all studies of Maori peoples) for population; as upper respiratory, lower respiratory, or unclear/both for outcome; as seasonal or meteorological parameters for exposure; and as a high-, middle-, or low-income country and by climate zone for location. When there are at least two effect sizes for each category, we will calculate a summary effect size.

Descriptive statistics will be conducted on each of the domains of the ROB. If enough data are available and ROB profiles vary, heterogeneity will be explored using sub-group analyses on each domain as an independent variable.

Publication bias is the concept that significant results are more likely to be published than non-significant research results [27]. An evaluation of publication bias will be conducted using a funnel plot (if *n* > 10 studies). Ratio measures of association will be plotted on the logarithmic scale to increase symmetry. A 95% confidence interval will be plotted, and different symbols will be used if heterogeneity is present. Interpretation of the funnel plot will be done visually. A test of significance for publication bias will be conducted using Egger’s test [28].

Finally, to summarize data about region of study and climate zone, descriptive spatial analyses will be performed. Specifically, point maps will be generated. The point map will illustrate specific study locations. When point locations are not provided in text with latitudinal and longitudinal coordinates, study sites will be geo-located using Google Maps^©^ and the best information available in the study. The point map will use open source shape files and will be built in R Spatial^©^ and R Maptools^©^ statistical packages.

## Strategy for presentation of the results

The final search strategy for each database and all ancillary searches conducted will be provided in the Additional file 1 of the final report. A flow chart, following the PRISMA guidelines [17], will be used to illustrate where citations were eliminated during screening and ancillary searches, including information about the rationale for exclusion in full article screening.

To illustrate the potential for publication bias and small study effects, a funnel plot will plot the effect estimates (horizontal axes) against the standard error (vertical axes) for each meta-analysis with *n* > 10 studies [29].

The results of this review will be provided via text and characteristics of studies tables in a published journal article. The tables will describe the seasonal or meteorological exposure, the outcome(s) measured, the direction and magnitude of association, and the period of study. Descriptive statistics (i.e., frequencies, proportions, missing data) will be provided as extensions of this table when appropriate or in narrative (i.e., proportion of studies with each ARI outcome).

Individual study results will be presented in forest plots [17]. If heterogeneity exists, separate forest plots will be used to illustrate results by strata. If the data are too limited (i.e., fewer than two studies with the same population, exposure, outcome, and region) or are heterogeneous, the results will be presented in a forest plot without a summary effect size.

A summary of findings table for key outcomes will be generated based on the Grading of Recommendations Assessment, Development and Evaluation [30]. A priori key outcomes will include prevalence of upper ARI and prevalence of lower ARI. Additional key outcomes identified in the systematic literature review will be documented as protocol amendments.

A table will be provided to summarize the findings of the ROB assessment. This table will follow the ROB presentation suggested in the PRISMA guidelines [17]. An additional column will highlight the rationale for the study’s ROB level.

Maps indicating the specific location of studies (point map) will be generated. Climate zones will be indicated on each map (e.g., tropical, temperate, or arctic).

## Ethical considerations

This research does not involve working directly with Indigenous and non-Indigenous communities, but rather with previous research conducted with these communities. In conducting this research, ethical principles will still be at the forefront, and will involve considerations for small population sizes and framing of the findings.

## Discussion

This systematic review protocol presents the method for the synthesis of current evidence related to differences in seasonal or meteorological association with ARI between Indigenous and non-Indigenous peoples living in the same region. This proposed review will likely be the first to summarize the potentially different associations between ARI and weather parameters between Indigenous and non-Indigenous peoples.

The results of the meta-analysis will examine whether Indigenous peoples are equally susceptible to associations between weather parameters and ARI, and whether this relationship varies by place, population, or other characteristics. A deeper understanding of this relationship will advance the academic literature and potentially lead to intervention strategies as climate change progresses. Further, an understanding of the differences between Indigenous and non-Indigenous communities can aid in planning, resource allocation, and determination of appropriate interventions.

## Additional file

Additional file 1: The Preferred Reporting Items for Systematic review and Meta-Analysis Protocols (PRISMA-P) 2015 checklist recommended items to address in a systematic review protocol with red text to demonstrate where information can be found in the body text. (DOCX 19 kb)

## Abbreviations

ARI: Acute respiratory infections
IWGIA: International Work Group for Indigenous Affairs
PECO: Population, exposure, comparator, and outcome
PRISMA: Preferred Reporting Items for Systematic Review and Meta-Analysis
ROB: Risk of bias
ROR: Ratio of odds ratios
UNHCR: United Nations Refugee Agency
