# Supplementary material for: A protocol for a systematic literature review: comparing the impact of seasonal and meteorological parameters on acute respiratory infections in Indigenous and non-Indigenous peoples
Source: Syst Rev. 2017 Jan 26;6:19. doi: 10.1186/s13643-016-0399-x (PMC5267362; doi:10.1186/s13643-016-0399-x)
Supplement: Additional file 1: — The Preferred Reporting Items for Systematic review and Meta-Analysis Protocols (PRISMA-P) 2015 checklist recommended items to address in a systematic review protocol with red text to demonstrate where information can be found in the body text. (DOCX 19 kb) [file 13643_2016_399_MOESM1_ESM.docx]

## Additional file 1

**The Preferred Reporting Items for Systematic review and Meta-Analysis Protocols (PRISMA-P) 2015 checklist recommended items to address in a systematic review protocol with red text to demonstrate where information can be found in the body text.**

| Section and topic | Item No | Checklist item |
| --- | --- | --- |
| ADMINISTRATIVE INFORMATION | | |
| Title: |  |  |
| Identification | 1a | Identify the report as a protocol of a systematic review (Page 1, Title) |
| Update | 1b | If the protocol is for an update of a previous systematic review, identify as such (N/A) |
| Registration | 2 | If registered, provide the name of the registry (such as PROSPERO) and registration number (N/A) |
| Authors: |  |  |
| Contact | 3a | Provide name, institutional affiliation, e-mail address of all protocol authors; provide physical mailing address of corresponding author (Page 1, Title Page) |
| Contributions | 3b | Describe contributions of protocol authors and identify the guarantor of the review (Page 19, Author’s Contributions) |
| Amendments | 4 | If the protocol represents an amendment of a previously completed or published protocol, identify as such and list changes; otherwise, state plan for documenting important protocol amendments (N/A) |
| Support: |  |  |
| Sources | 5a | Indicate sources of financial or other support for the review (Page 19, Funding and Acknowledgements) |
| Sponsor | 5b | Provide name for the review funder and/or sponsor (Page 19, Funding and Acknowledgements) |
| Role of sponsor or funder | 5c | Describe roles of funder(s), sponsor(s), and/or institution(s), if any, in developing the protocol (N/A) |
| INTRODUCTION | | |
| Rationale | 6 | Describe the rationale for the review in the context of what is already known (Page 3, Background) |
| Objectives | 7 | Provide an explicit statement of the question(s) the review will address with reference to participants, interventions, comparators, and outcomes (PICO) (Page 4, Review Question; Page 5, Participants Eligible; Page 5, Exposures Eligible; N/A; Page 5, Outcome Measures Eligible) |
| METHODS | | |
| Eligibility criteria | 8 | Specify the study characteristics (such as PICO, study design, setting, time frame) and report characteristics (such as years considered, language, publication status) to be used as criteria for eligibility for the review (Page 4, Study Designs Eligible; Page 7-10, Search Methods for the Identification of Studies; Pages 10-11, Selection of Eligible Studies) |
| Information sources | 9 | Describe all intended information sources (such as electronic databases, contact with study authors, trial registers or other grey literature sources) with planned dates of coverage (Page 7-10, Search Methods for the Identification of Studies) |
| Search strategy | 10 | Present draft of search strategy to be used for at least one electronic database, including planned limits, such that it could be repeated (Page 23-31, Table 1A-C) |
| Study records: |  |  |
| Data management | 11a | Describe the mechanism(s) that will be used to manage records and data throughout the review (Pages 11-13, Data Collection from Eligible Studies; Pages 13-14, Process for Data Extraction) |
| Selection process | 11b | State the process that will be used for selecting studies (such as two independent reviewers) through each phase of the review (that is, screening, eligibility and inclusion in meta-analysis) (Pages 10-11, Selection of Eligible Studies) |
| Data collection process | 11c | Describe planned method of extracting data from reports (such as piloting forms, done independently, in duplicate), any processes for obtaining and confirming data from investigators (Pages 11-13, Data Collection from Eligible Studies; Pages 13-14, Process for Data Extraction) |
| Data items | 12 | List and define all variables for which data will be sought (such as PICO items, funding sources), any pre-planned data assumptions and simplifications (Pages 11-13, Data Collection from Eligible Studies; Page 14, Risk-of-Bias Assessment for Eligible Studies; Page 15, Confounders Relevant to All or Most Studies) |
| Outcomes and prioritization | 13 | List and define all outcomes for which data will be sought, including prioritization of main and additional outcomes, with rationale (Page 5, Outcome Measures Eligible; Pages 11-13, Data Collection from Eligible Studies) |
| Risk of bias in individual studies | 14 | Describe anticipated methods for assessing risk of bias of individual studies, including whether this will be done at the outcome or study level, or both; state how this information will be used in data synthesis (Page 14, Risk-of-Bias Assessment for Eligible Studies) |
| Data synthesis | 15a | Describe criteria under which study data will be quantitatively synthesised (Pages 15-17, Strategy for Data Synthesis) |
|  | 15b | If data are appropriate for quantitative synthesis, describe planned summary measures, methods of handling data and methods of combining data from studies, including any planned exploration of consistency (such as I^2^, Kendall’s τ) (Pages 15-17, Strategy for Data Synthesis; Page 17-18, Strategy for Presentation of the Results) |
|  | 15c | Describe any proposed additional analyses (such as sensitivity or subgroup analyses, meta-regression) (Pages 15-17, Strategy for Data Synthesis; Page 14, Risk-of-Bias Assessment for Eligible Studies) |
|  | 15d | If quantitative synthesis is not appropriate, describe the type of summary planned (N/A) |
| Meta-bias(es) | 16 | Specify any planned assessment of meta-bias(es) (such as publication bias across studies, selective reporting within studies) (Pages 15-17, Strategy for Data Synthesis) |
| Confidence in cumulative evidence | 17 | Describe how the strength of the body of evidence will be assessed (such as GRADE) (Page 14, Risk-of-Bias Assessment for Eligible Studies) |

*** It is strongly recommended that this checklist be read in conjunction with the PRISMA-P Explanation and Elaboration (cite when available) for important clarification on the items. Amendments to a review protocol should be tracked and dated. The copyright for PRISMA-P (including checklist) is held by the PRISMA-P Group and is distributed under a Creative Commons Attribution Licence 4.0.**

*From: Shamseer L, Moher D, Clarke M, Ghersi D, Liberati A, Petticrew M, Shekelle P, Stewart L, PRISMA-P Group. Preferred reporting items for systematic review and meta-analysis protocols (PRISMA-P) 2015: elaboration and explanation. BMJ. 2015 Jan 2;349(jan02 1):g7647.*
